# Retinoic Acid Signalling Regulates Zebrafish Tooth Germ Repair Following Injury

**DOI:** 10.1111/cpr.70186

**Published:** 2026-02-23

**Authors:** Qiqi Liu, Zhenan Zhang, Weifeng Hao, Chunyan Zhou, Deqin Yang

**Affiliations:** ^1^ Department of Endodontics The Affiliated Stomatological Hospital of Chongqing Medical University Chongqing China; ^2^ Chongqing Key Laboratory of Oral Diseases Chongqing China; ^3^ Chongqing Municipal Key Laboratory of Oral Biomedical Engineering of Higher Education Chongqing China; ^4^ Chongqing Municipal Health Commission Key Laboratory of Oral Biomedical Engineering Chongqing China; ^5^ Department of Conservative Dentistry and Endodontics Shanghai Stomatological Hospital & School of Stomatology, Fudan University Shanghai China; ^6^ Shanghai Key Laboratory of Craniomaxillofacial Development and Diseases, Fudan University Shanghai China

**Keywords:** retinoic acid signalling, SCPP5, tooth germ injury, tooth germ repair, zebrafish

## Abstract

Although the role of retinoic acid (RA) signalling in odontogenesis is well established, its involvement in the repair of injured tooth germs remains unclear. To investigate this, we generated a *Tg*(*scpp5:Dendra2‐NTR*) zebrafish line for labelling tooth germ cells and established a tooth germ injury model using the nitroreductase (NTR)/metronidazole (MTZ) system. We then modulated RA signalling by exogenous activation with RA, retinol, retinal, talarozole (TZ) or *Tg*(*hsp70l:aldh1a2‐p2a‐mCherry; cryaa:venus*), and by suppression with 4‐diethylaminobenzaldehyde (DEAB) or *Tg*(*hsp70l:dnRARAA‐p2a‐DsRed; cryaa:venus*), to examine its function in tooth germ repair. Following targeted ablation of tooth germ cells, RA signalling was activated, with *aldh1a2* showing the most pronounced upregulation. Exogenous RA promoted injury‐induced tooth germ repair, whereas its precursors (retinol and retinal) had no significant effect on aldh1a2 expression or repair. Pharmacological inhibition of RA degradation with TZ enhanced repair, while dominant‐negative inhibition of RA signalling impaired it. Furthermore, modulation of *aldh1a2* revealed its essential role: inhibition with DEAB attenuated repair, whereas genetic activation facilitated tissue restoration. In summary, this study clarifies the regulatory role of RA signalling in tooth germ injury repair, offering a theoretical foundation and potential therapeutic targets for the treatment of injured tooth germs.

## Graphical abstract

Retinoic acid signalling may regulate the repair processes in a tooth germ injury model using *Tg*(*scpp5:Dendra2‐NTR*) zebrafish and the nitroreductase (NTR)/metronidazole (MTZ) system.

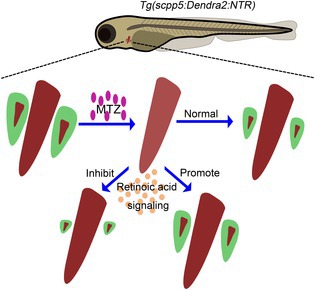

## 1 Introduction

The development of human tooth, from tooth germ initiation to mineralisation and maturation, is a complex process driven by epithelial–mesenchymal interactions [1]. However, with limited ability for self‐repair, disruptions from genetic or environmental factors can impair tooth germ development, leading to a high prevalence of damage in dental hard tissues worldwide [2]. Current clinical treatments for these tooth injuries rely primarily on adhesive or fixed restorations, all of which rely on the use of synthetic materials. Therefore, re‐examining tooth germ repair following injury from a molecular biology perspective is a promising approach. The zebrafish pharyngeal dentition consists of three distinct rows—ventral, mediodorsal and dorsal [3]. The first tooth initiates development at 48 hpf (hours post‐fertilisation) at position 4 in the ventral row (designated 4V1, where ‘1’ denotes the generation number) [4]. Unlike the enamel‐dentine interface of mammals, the mineralised surface of zebrafish teeth consists of enameloid, an enamel‐like primitive hypermineralised tissue [5]. The ontogeny of zebrafish teeth unfolds through five spatiotemporally coordinated stages: initiation, morphogenesis, early cytodifferentiation, late cytodifferentiation and attachment [4, 6, 7]. The 4V1 tooth achieves functional attachment to the fifth ceratobranchial by 96 hpf [4]. Critically, the zebrafish teeth undergo lifelong renewal through sequential regeneration, a process mechanistically analogous to diphyodont replacement in certain mammals [8]. This unique trait establishes zebrafish as a powerful model for studying post‐injury tooth germ repair.

The secretory calcium‐binding phosphoprotein (SCPP) gene family plays a pivotal role in the formation of both dental and skeletal tissues [9, 10, 11]. Phylogenetic analysis reveals the SCPP gene family diverges into two functionally specialised subgroups—acidic SCPPs characterised by clustered phosphorylation sites and P/Q‐rich SCPPs containing repetitive proline‐glutamine motifs [12]. As a member of the P/Q‐rich SCPP gene family, *scpp5* demonstrates unique cell‐type specificity in zebrafish, being expressed solely in the odontogenic lineage (inner dental epithelium and odontoblasts) but undetectable in osteogenic cells (osteoblasts/osteocytes) [13].

The nitroreductase (NTR)/metronidazole (MTZ) system combines precise spatial control (via cell‐type‐specific NTR expression) with temporal regulation (through MTZ administration) [14]. NTR‐mediated reduction of MTZ generates DNA‐alkylating nitroso intermediates that induce targeted cell death while sparing neighbouring NTR‐negative populations [15, 16]. During early postnatal development (≤ 7 days post‐fertilisation, dpf), zebrafish dentition comprises only the first two tooth generations located in the 3V, 4V and 5V positions [4]. This sparse tooth distribution allows for easy individual identification and observation of each tooth germ. Taking advantage of this morphological simplicity and the odontogenic lineage‐specific expression of *scpp5*, we employed the NTR/MTZ system to achieve spatiotemporal ablation of tooth germ cells, thereby establishing a precise tooth germ injury model.

Retinoic acid (RA) refers to synthetic or natural derivatives of retinol (vitamin A) and is essential for the development of chordate embryos [17]. It acts in a localised manner, functioning through both cell‐autonomous (autocrine) and non‐cell‐autonomous (paracrine) mechanisms to influence either the producing cell itself or adjacent target cells [18]. Hepatic retinol is mobilised by binding to retinol‐binding protein (RBP) and is subsequently distributed systemically via the circulatory system [19]. In plasma, retinol circulates as a stoichiometric complex with retinol‐binding protein 4 (RBP4) and transthyretin (TTR) [20]. However, unlike the TTR–RBP4–retinol ternary complex found in mammals, zebrafish utilise a TTR‐independent pathway for retinol transport [21]. Target cells take up retinol through either: (i) active transport via the stimulated by retinoic acid 6 (STRA6) transmembrane channel, or (ii) passive membrane diffusion [22]. Upon entering the cytosol, retinol is immediately bound by cellular retinol‐binding protein 1 (CRBP1), which facilitates its intracellular trafficking [23]. Intracellular retinol can be metabolically converted into retinyl esters via lecithin retinol acyltransferase (LRAT)‐catalysed esterification, forming stable storage compounds within cytoplasmic lipid droplets [24]. Alternatively, retinol can be oxidised in two steps to generate RA [19]. The first step, oxidation to retinal (retinaldehyde), is primarily catalysed by retinol dehydrogenase 10 (RDH10). Alcohol dehydrogenase (ADH) can also catalyse this conversion, but only when retinol is not bound to CRBP1 [25]. The reverse reaction—reduction of retinal back to retinol—is mediated by dehydrogenase/reductase 3 (DHRS3) [26, 27]. The second and irreversible step, catalysed by retinaldehyde dehydrogenases (RALDHs), converts retinal into RA [28]. The cytochrome P450 26 (CYP26) enzyme subfamily mediates the oxidative degradation of excess RA, preventing its potentially harmful accumulation through metabolic clearance [29]. In addition to paracrine signalling, RA also functions in autocrine signalling via cellular retinoic acid‐binding protein 2 (CRABP2)‐mediated translocation into the nucleus [30]. RA serves as the primary ligand for nuclear retinoic acid receptors (RARs) [17]. Upon binding, RARs form heterodimers with retinoid X receptors (RXRs) and bind to retinoic acid response elements (RAREs), where they recruit co‐regulators to modulate the transcription of target genes [31, 32].

Studies showed that RA is required for induction of pharyngeal teeth in zebrafish [33, 34]. Disruption of RA signalling abolished tooth formation while maintaining normal development of the associated ceratobranchial [35]. Among the three CYP26 subfamily members (A1/B1/C1), heterozygous *cyp26b1* mutant induced the formation of an extra tooth in the ventral row of adult zebrafish [36]. Moreover, exogenous RA treatment in zebrafish embryos induced expansion of tooth‐forming fields, accompanied by extended expression domains of pharyngeal mesenchymal markers (*dlx2a*, *lhx6*) and dental epithelial marker *pitx2a* [36, 37]. While zebrafish naturally develop pharyngeal teeth but lack oral dentition, pharmacological inhibition of endogenous RA degradation can induce ectopic oral tooth formation [38]. However, the role of RA signalling in post‐injury dental repair remains enigmatic.

In this paper, we developed a tooth germ injury model in *Tg*(*scpp5:Dendra2‐NTR*) zebrafish using NTR/MTZ system. The activation of RA signalling was detected post‐MTZ removal. Using small‐molecule perturbations or targeted genetic manipulations, we next dissected the spatiotemporal requirements of RA signalling in post‐injury repair. The data lead to the conclusion that modulation of RA signalling exerts bidirectional effects on tooth germ injury repair—upregulation promotes while downregulation inhibits the repair process, with both RA and raldh1a2 serving as critical roles.

## 2 Materials and Methods

Zebrafish of the AB genetic background were used as WT zebrafish. The environmental conditions of the zebrafish fish room were standard laboratory conditions: temperature 28.5°C; photoperiod 14‐h light cycle/10‐h dark cycle. All experiments were performed following the guidelines of ARRIVE (Animal Research: Reporting of In Vivo Experiments). The experimental protocols were approved by the Ethics Committee of Stomatological Hospital of Chongqing Medical University (Approval No. 2020022).

### 2.1 Generation of Plasmid and Transgenic Line

To construct the *pBluescript‐scpp5:Dendra2‐NTR* plasmid, the 4900‐bp promoter of *scpp5* was cloned from zebrafish genomic DNA, and the following primer sequences are used for PCR amplification: *scpp5*‐F: 5′‐AGGGGGCCCCCTCGAAGATCGAGACCGTAGCA‐3′; *scpp5*‐R: 5′‐CCCACCGGTGCCGGAGAGACGTCCACATGGTT‐3′. These promoter sequences were subcloned into *pBluescript‐Dendra2‐NTR* vector between the *Apa*I(NEB) and *Age*I(NEB) enzyme sites. The *pBluescript‐scpp5:Dendra2‐NTR* plasmid was co‐injected with I‐SceI(NEB) into the one‐cell stage of embryos under the AB genetic background for transgenesis. The transgenic line was outcrossed at least every other generation to ensure genetic diversity.

### 2.2 Drug Treatment and Heat Shock Conditions

MTZ (MCE) was dissolved in egg water containing 0.003% PTU and 0.2% DMSO, and stored protected from light at the final concentration. Zebrafish were treated with MTZ at 3 dpf for 48 h and incubated in the egg water with 0.003% PTU. For small molecule treatment, zebrafish were incubated in egg water not only containing 0.003% PTU and 0.2% DMSO, but also containing 10^−8^ M RA (all‐trans‐Retinoic acid; MCE), 10^−6^ retinal (all‐trans‐Retinal; MCE), 10^−6^ M retinol (all‐trans‐Retinol; MCE), 10^−6^ M talarozole (TZ/R115866; MCE) or 10^−5^ M 4‐diethylaminobenzaldehyde (DEAB; MCE). To ensure consistent pharmacological activity, the drug solutions were refreshed every 24 h.

Zebrafish of *Tg*(*hsp70l:dnRARAA‐p2a‐DsRed; cryaa:venus*) and *Tg*(*hsp70l:aldh1a2‐p2a‐mCherry; cryaa:venus*) were heat‐shocked at 38.5°C for 40 min at the indicated stage, then incubated at 28.5°C, with heat shock performed every 12 h.

### 2.3 In Situ Hybridisation (ISH)

Embryos were collected at the corresponding time points and fixed overnight in 4% paraformaldehyde (PFA). After removing the PFA, the embryos were washed several times. The primers used for the probes in the in situ hybridisation assay are listed in Table S1. The prepared probes were hybridised overnight in a 68.5°C water bath. After recovering the probes, embryos were washed in gradient solutions of SSCT and MABT. Anti‐Dig‐AP antibody (Roche) was added, and the embryos were incubated overnight on a shaking platform at 4°C. After removing the antibody, the embryos were washed several times with MABT. The embryos were then stained in the dark at 37°C in a solution of BCIP dissolved in NTMT. After staining was completed, the reaction was stopped by adding a stop solution (containing 0.05 μM phosphate‐buffered saline, 1 mM EDTA and 0.1% Tween‐20). The stained embryos were observed under a microscope (Carl Zeiss).

### 2.4 Antibody Staining

Embryos fixed overnight in 4% PFA were washed several times with PBST, and then the ventral pharyngeal skin, heart, liver, yolk and other tissues were carefully removed using tissue forceps under a microscope. The embryos were then treated with acetone at −30°C for 30 min. After washing several times with PT, the primary antibody (Dendra2, 1:1000; Antibodies‐online, ABIN361314) was incubated overnight at 4°C. After removing the primary antibody, the embryos were washed several times with PT. The secondary antibody (Alexa Fluor 488, 1:1000; Invitrogen) was incubated overnight at 4°C. After removing the secondary antibody, the embryos were washed several times in the dark with PT. Images were captured using a Carl Zeiss LSM780 laser scanning confocal microscope.

### 2.5 Fluorescence In Situ Hybridisation (FISH)

Embryos were treated with 3% hydrogen peroxide in the dark for 1 h, followed by several washes with PBST at room temperature. Using tissue forceps under a microscope, the ventral pharyngeal skin, heart, liver, yolk and other tissues were carefully removed. The embryos were then washed several times with PBST. The probe was hybridised overnight in a 65°C water bath, with the primers used listed in Table S1. Anti‐Dig‐POD (Roche) was incubated overnight at 4°C, followed by several washes with PT. The embryos were then incubated with Cy3 (Roche) at room temperature overnight. The subsequent steps followed the antibody staining procedure as previously described.

### 2.6 TUNEL Staining

After the sample collection was completed, embryos were fixed with 4% PFA. TUNEL staining was performed according to the instructions provided with the TUNEL kit (Roche). After staining, embryos were incubated with primary and secondary antibodies following the antibody staining procedure. Images were captured using a Carl Zeiss LSM780 laser scanning confocal microscope. The relative number of TUNEL^+^ cells was calculated as the ratio of the number of apoptotic cells to the Dendra2 fluorescent intensity.

### 2.7 Alizarin Red Staining

The embryo handling process was the same as described previously for antibody staining. After removing the skin, heart, liver, yolk and other tissues under a microscope, embryos were dehydrated in 50% ethanol at room temperature for 10 min. After removing the 50% ethanol, the embryos were immersed in a 0.05% Alizarin Red staining solution (Beyotime, ST1078) and placed in the dark on a shaking platform overnight. After staining, embryos were treated with a bleaching solution (final concentration of 1.5% H_2_O_2_ and 1% KOH) at room temperature, with the tube cap left open for 20 min. Finally, embryos were incubated in a solution composed of 20% glycerol and 0.25% KOH at room temperature overnight. The embryos were then photographed under a Carl Zeiss LSM780 laser scanning confocal microscope.

### 2.8 Quantitative Polymerase Chain Reaction (qPCR)

For each treatment group, the ceratobranchial regions from 200 zebrafish were collected at the respective time points. Total RNA was extracted from these tissues using NucleoZOL reagent (MACHEREY NAGEL) according to the manufacturer's instructions, and then reverse transcribed into cDNA using a reverse transcription kit (Bioground, BG0070). qPCR was performed using SYBR reagent (Bioground, BG0014). The primer sequences are shown in Table S2. GAPDH was used as the housekeeping gene, and three independent experiments were conducted.

### 2.9 Statistical Analysis

Statistical analysis was performed using GraphPad Prism 9 software and error bars represent the standard error of the mean (SEM). The intensities of the fluorescence images and relative TUNEL^+^ cells were measured through ZEN 2012 (ZEISS). The Student's *t*‐test was used to compare two independent samples. In studies with multiple independent groups, one‐way ANOVA is used to examine the effect of a single independent variable, while two‐way ANOVA is used to assess the effects of two independent variables. Tukey was subsequently applied for multiple comparisons in one‐way and two‐way ANOVA models. A *p* value of < 0.05 was considered statistically significant.

## 3 Results

### 3.1 Establishment of a Tooth Germ Model Based on Transgenic Zebrafish Line *Tg*(*scpp5:Dendra2‐NTR*)

**FIGURE 1:**
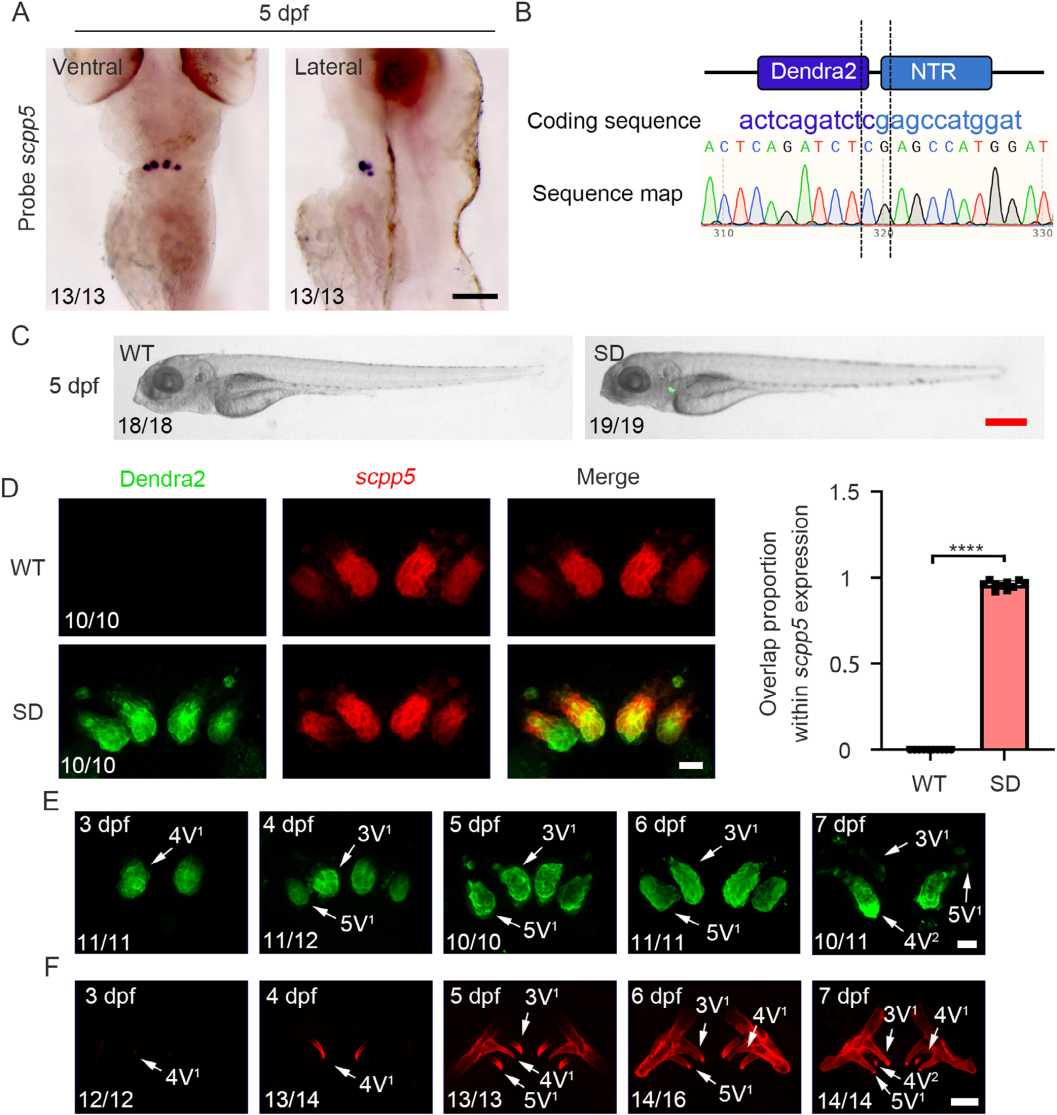
Establishment of a tooth germ marker model based on *Tg*(*scpp5:dendra2‐NTR*) zebrafish transgenic line. In situ hybridisation (ISH) showing the expression pattern of the *scpp5* probe. Scale bar is 100 μm (A). Sequencing results of the transgenic line. The dashed lines indicate the Dendra2‐NTR sequence (B). Stereomicroscopy images of transgenic and wild‐type (WT) zebrafish. Scale bar is 500 μm (C). Fluorescence in situ hybridisation (FISH) showing co‐localisation of the *scpp5* probe (red) and Dendra2 protein (green). Scale bar is 20 μm (D). Anti‐Dendra2 staining showing fluorescence in *Tg*(*scpp5:dendra2‐NTR*) transgenic line from 3 to 7 days post‐fertilisation (dpf). Scale bar is 20 μm (E). Alizarin red staining showing zebrafish tooth from 3 to 7 dpf. Scale bar is 50 μm (F). 3V^1^, The first generation‐tooth at position 3 in the ventral row; dpf, days post‐fertilisation; SD, scpp5:dendra2‐NTR; WT, Wild Type. ****p < 0.0001.

To establish a tooth mineralisation marker model, we first examined *scpp*5 expression in zebrafish at 5 dpf using in situ hybridisation (ISH). *Scpp5* was specifically expressed in the pharyngeal tooth germ region, but not in surrounding tissues, indicating that the scpp5 probe could precisely localise and visualise tooth germs (Figure 1A). We then cloned a 4.9 kb fragment upstream of the scpp5 translation start site from the zebrafish genome and integrated it into a vector containing Dendra2‐NTR, generating the recombinant plasmid *scpp5:Dendra2‐NTR*. Genomic sequencing confirmed correct plasmid insertion into the zebrafish genome (Figure 1B). Embryos injected with this plasmid and fluorescence was specifically observed in the tooth region under a stereomicroscope, with no fluorescence detected in wild‐type zebrafish (AB strain) (Figure 1C). Fluorescence in situ hybridisation (FISH) revealed co‐localisation of green fluorescence (Anti‐Dendra2) and red fluorescence (*scpp5* probe), confirming that Dendra2‐positive cells were indeed scpp5 expressing cells (Figure 1D). We next observed tooth germ development in the *Tg*(*scpp5:Dendra2‐NTR*) between 3 and 7 dpf (Figure 1E,F). Antibody staining revealed that 4V1 fluorescence was detectable only at 3 dpf, consistent with prior reports that *scpp5* expression in 4V1 is restricted to late cytodifferentiation (Figure 1E) [39]. In both 3V1 and 5V1, fluorescent labelling became visible starting at 4 dpf, exhibited a gradual rise in intensity peaking at 6 dpf and then displayed obvious weakening by 7 dpf (Figure 1E). Alizarin red staining showed 4V1 ceratobranchial attachment at 4 dpf, followed by 3V1/5V1 attachment at 6 dpf and 4V2 emergenced at 7 dpf (Figure 1F). These results indicate that the transgenic zebrafish line *Tg*(*scpp5:Dendra2‐NTR*) has been successfully established.

### 3.2 A Tooth Germ Injury Model Is Established in *Tg*(*scpp5:Dendra2‐NTR*) by NTR/MTZ System

**FIGURE 2:**
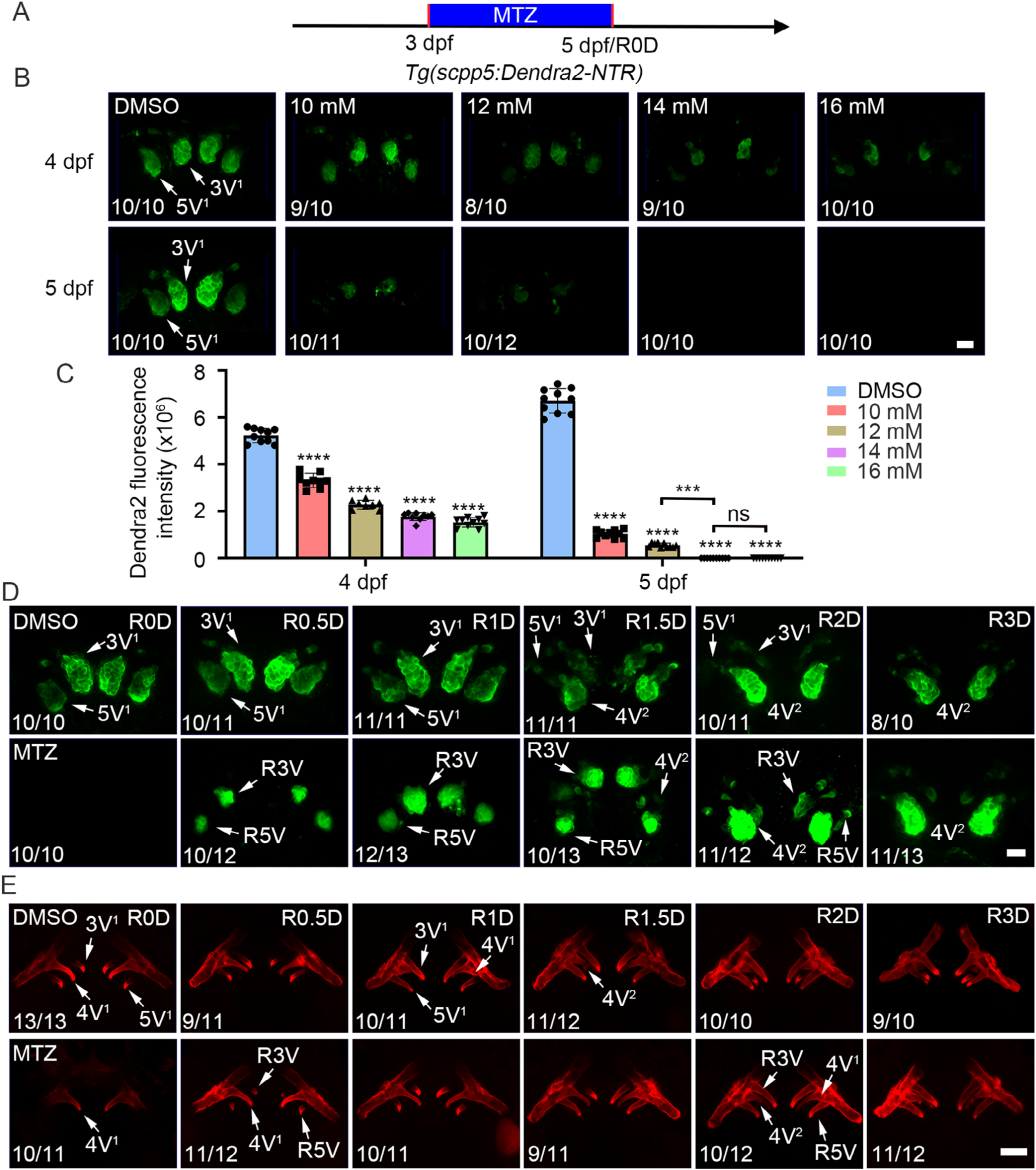
Tooth germs in *Tg*(*scpp5:Dendra2‐NTR*) exhibit robust repair following injury. Experimental schedule of MTZ injury (A). Antibody staining showing the effect of different MTZ on the Dendra2 fluorescence. Scale bar is 20 μm (B, C). Antibody staining showing the Dendra2 fluorescence in DMSO and MTZ‐treated groups during the repair process. Scale bar is 20 μm (D). Alizarin red staining showing the tooth in DMSO and MTZ‐treated groups during the repair process. Scale bar is 50 μm (E). 3V^1^, The first generation‐tooth at position 3 in the ventral row; dpf, days post‐fertilisation; MTZ, Metronidazole; R0D, 0 day of repair; R3V, The repair‐tooth at position 3 in the ventral row. ns no significance, ***p < 0.001 and ****p < 0.0001.

After successfully establishing the *Tg*(*scpp5:Dendra2‐NTR*) zebrafish, we tested various concentrations of MTZ to induce tooth germ injury. At MTZ concentrations ≥ 14 mM, Dendra2‐labelled dental germ cells were completely eliminated, as shown by antibody staining (Figure 2A–C). Additionally, zebrafish survival remained unaffected by 10–16 mM MTZ treatment at R0D (0 day of repair) (Figure S1A). Therefore, in subsequent experiments, we treated zebrafish with 14 mM MTZ at 3–5 dpf to establish a tooth germ injury model. Given that the NTR/MTZ system was linked to the accumulation of apoptotic cells, TUNEL staining was employed to confirm that NTR‐expressing tooth germ cells underwent apoptotic cell death (Figure S1B–D) [40, 41].

**FIGURE 3:**
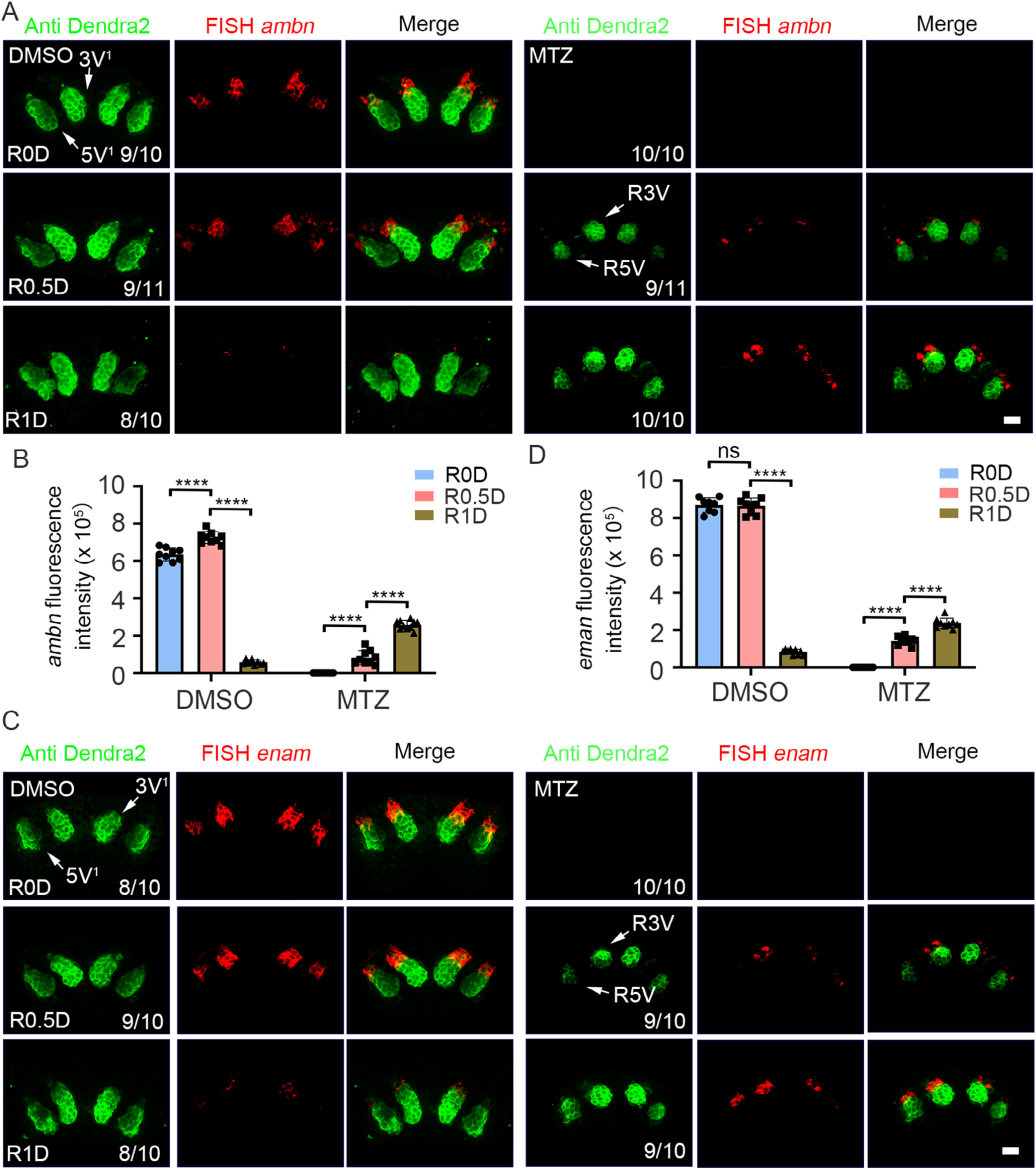
Expression patterns of *ambn* and *enam* during the early stage of tooth germ repair. FISH for *ambn* in DMSO and MTZ‐treated groups from R0D to R1D. Scale bar is 20 μm (A, B). FISH for *enam* in DMSO and MTZ‐treated groups from R0D to R1D. Scale bar is 20 μm (C, D). 3V^1^, The first generation‐tooth at position 3 in the ventral row; MTZ, Metronidazole; R0D, 0 day of repair; R3V, The repair‐tooth at position 3 in the ventral row. ns no significance and ****p < 0.0001.

Antibody staining and alizarin red staining were used to monitored the repair process of tooth germs from R0D to R3D post‐MTZ treatment (Figure 2D,E). In the MTZ group, only the 4V1 was present at R0D, while both 3V1 and 5V1 were absent. During repair, R3V/R5V (repair‐tooth at position 3 in the ventral row/repair‐tooth at position 5 in the ventral row) attached to the ceratobranchial by R2D, with fluorescence peaking at R1D‐R1.5D and declining post‐R1.5D. In the DMSO group, 3V1/5V1 attachment occurred earlier (R1D), followed by fluorescence decay (Figure 2D,E). 4V2 appeared later in the MTZ group versus DMSO group (Figure 2D,E). From R0D to R3D, ISH was performed to examine the expression of zebrafish tooth germ markers, *fth1b* and *cx43* [42, 43]. In the MTZ‐treated group, the expression domains of both markers gradually expanded from R0D to R1D (Figure S2A,B). Furthermore, FISH analysis revealed that the expression of tooth mineralisation‐related genes, *ambn* and *enam* [44], progressively increased in MTZ group between R0D and R1D, whereas the expression *ambn* and *enam* was decreased in DMSO group at R1D (Figure 3A–D). Collectively, our findings establish *Tg*(*scpp5:Dendra2‐NTR*) zebrafish as a model for tooth germ injury, enabled by the targeted cell ablation of the NTR/MTZ system.

### 3.3 Activation of Retinoic Acid Signalling Following Injury

**FIGURE 4:**
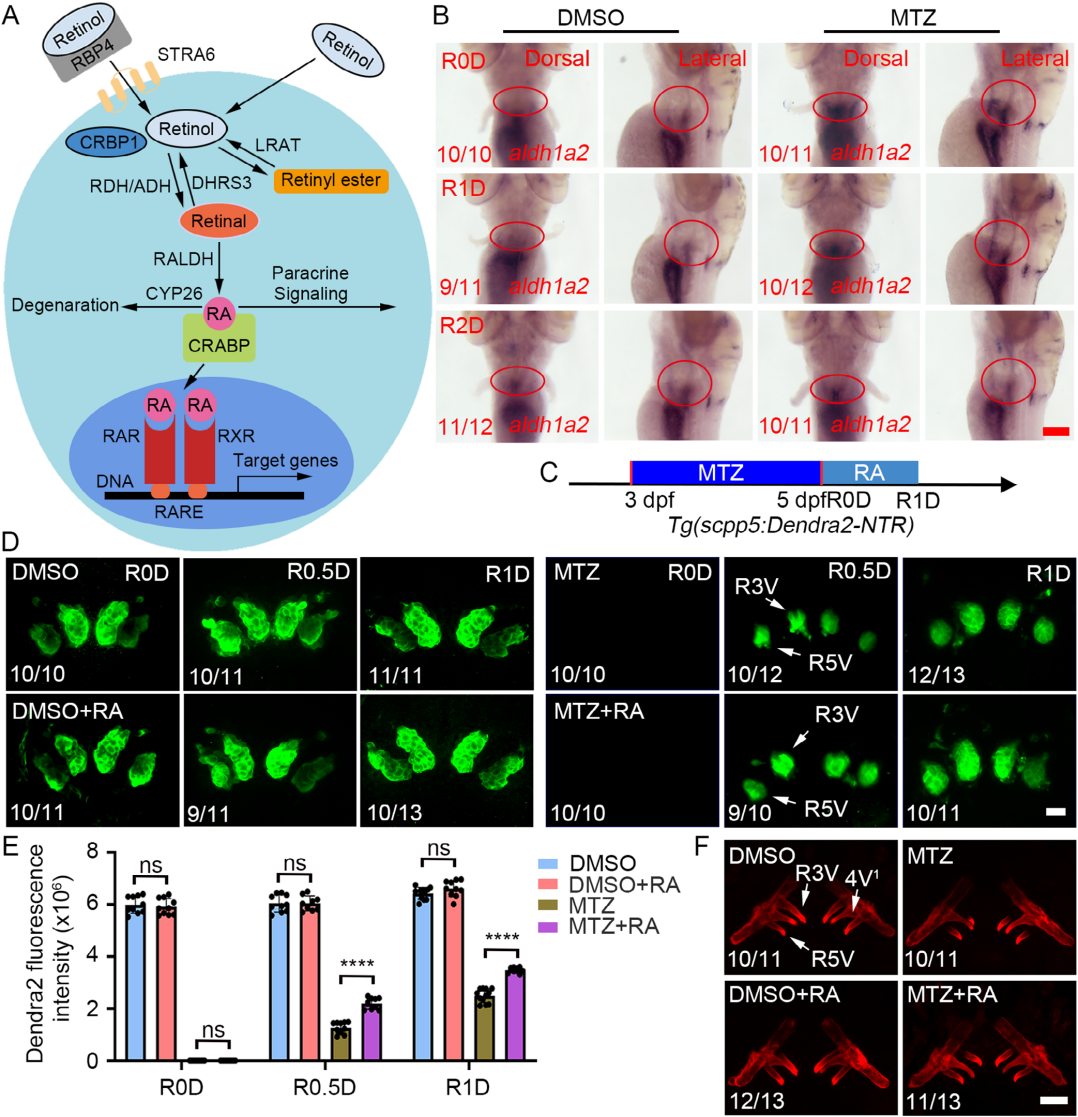
Injury‐induced RA signalling activation and exogenous RA enhance tooth repair. Schematic diagram of RA signalling (A). ISH showing the expression of *aldh1a2* in DMSO and MTZ‐treated groups at R0D, R1D and R2D. Scale bar is 100 μm (B). Experimental schedule of exogenous RA treatment (C). Antibody staining showing the Dendra2 fluorescence in DMSO, MTZ, DMSO+RA and MTZ + RA groups from ROD to R1D. Scale bar is 20 μm (D, E). Alizarin red staining showing the tooth in DMSO and MTZ + RA groups at R1D. Scale bar is 50 μm (F). 4V^1^, The first generation‐tooth at position 4 in the ventral row; ADH, alcohol dehydrogenase; CRABP, cellular retinoic acid‐binding protein; CRBP1, cellular retinol‐binding protein 1; CYP26, cytochrome P450 subfamily 26; DHSR3, dehydrogenase/reductase 3; dpf, days post‐fertilisation; LRAT, lecithin retinol acyltransferase; MTZ, Metronidazole; R0D, 0 day of repair; R3V, The repair‐tooth at position 3 in the ventral row; RA, retinoic acid; RALDH, retinaldehyde dehydrogenase; RAR, retinoic acid receptors; RARE, retinoic acid response element; RXR, retinoid X receptors; RBP4, retinol‐binding carrier protein 4; RDH, retinol dehydrogenase; STRA6, stimulated by retinoic acid 6. ns no significance and ****p < 0.0001.

Intracellularly, the main events of the RA signalling included retinol processed into retinal and further into RA (Figure 4A) [22]. To determine the functional involvement of RA signalling in post‐injury tooth germ repair, we quantified the expression of RA signalling‐related genes in zebrafish ceratobranchial region using qPCR (quantitative polymerase chain reaction) analysis. The genes adh1, raldh1 and rarb in the RA signalling pathway are absent in zebrafish. At R0D, qPCR analysis demonstrated that MTZ treatment significantly upregulated 18 retinoid pathway genes (*rbp4*, *stra6*, *rbp1.1*, *lratb.1*, *lratb.2*, *rdh10a*, *dhrs3a*, *dhrs3b*, *aldh1a2*, *crabp2a*, *crabp2b*, *cyp26b1*, *raraa*, *rarab*, *rarga*, *rxrab*, *rxrba* and *rxrbb*), while 7 genes (*rbp1.2*, *rdh10b*, *aldh1a3*, *crabp1a*, *crabp1b*, *rargb* and *rxraa*) showed no significant change and 5 genes (*lrata*, *cyp26a1*, *cyp26c1*, *rxrga* or *rxrgb*) remained undetectable in the ceratobranchial region (Figure S2C). Notably, *aldh1a2* exhibited the most pronounced upregulation among all RA signalling genes (8.6‐fold increase vs. DMSO, *p* < 0.001) (Figure S2C). ISH showed that MTZ treatment expands the domain of expression of *aldh1a2* at both R0D and R1D, while expression patterns remained unchanged at R2D (Figure 4B). Notably, these data reveal that MTZ‐induced injury triggers the activation of RA signalling.

### 3.4 RA, but Not Its Synthetic Precursors (Retinol or Retinal), Effectively Regulate Injury‐Induced Tooth Germ Repair

**FIGURE 5:**
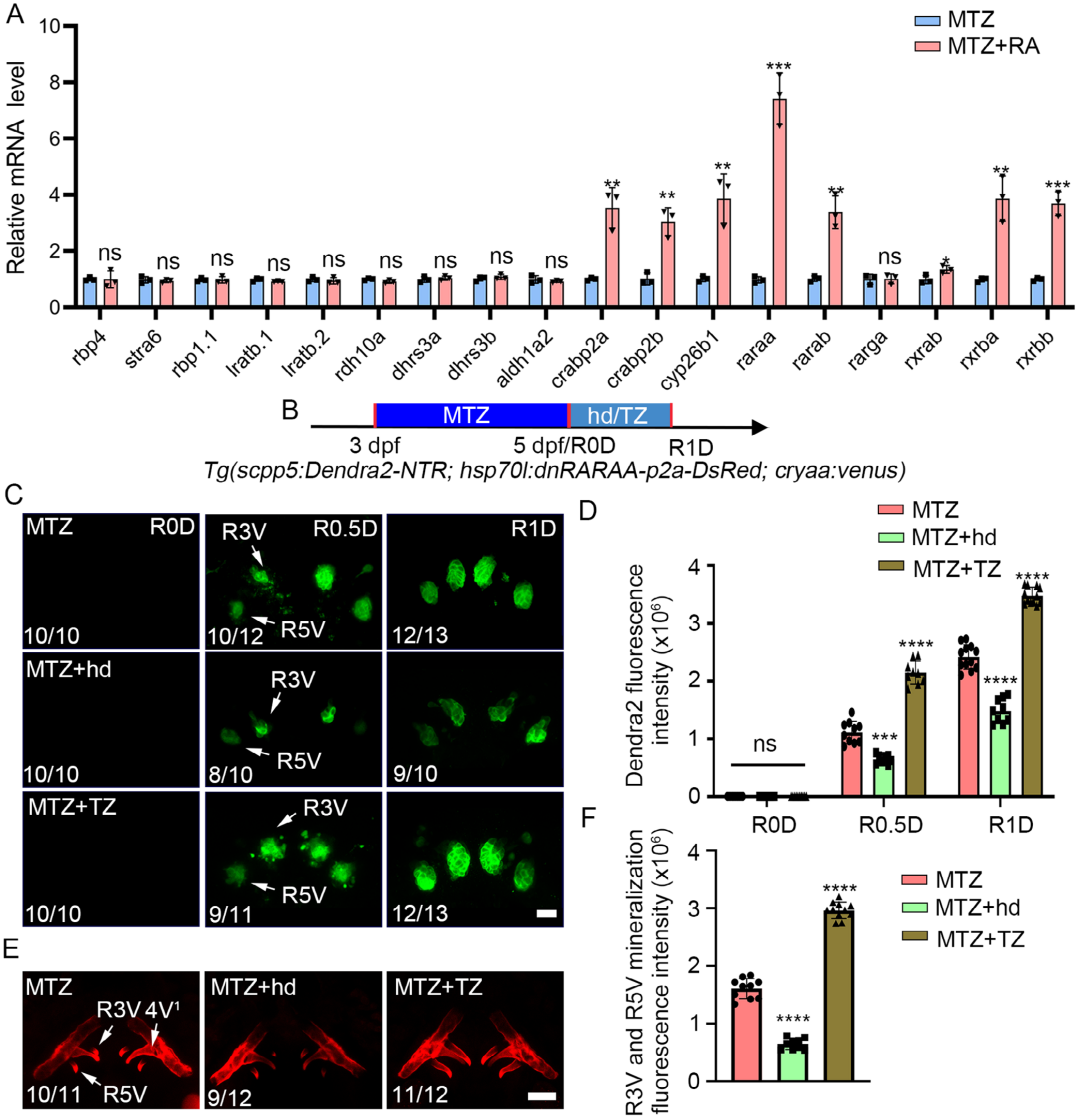
Modulating endogenous RA signalling function in tooth repair. qPCR showing the expression of the 18 retinoid pathway genes upregulated by MTZ (A). Experimental schedule of modulating endogenous RA signalling (B). Antibody staining showing the Dendra2 fluorescence in MTZ, MTZ + hd and MTZ + TZ groups from ROD to R1D. Scale bar is 20 μm (C, D). Alizarin red staining showing the tooth in MTZ, MTZ + hd and MTZ + TZ groups at R1D. Scale bar is 50 μm (E, F). 4V^1^, The first generation‐tooth at position 4 in the ventral row; dpf, days post‐fertilisation; R0D, 0 day of repair; R3V, The repair‐tooth at position 3 in the ventral row; TZ, talarozole; hd, *Tg*(*hsp70l:dnRARAA‐p2a‐DsRed; cryaa:venus*); MTZ, Metronidazole. ns no significance, ***p < 0.001 and ****p < 0.0001.

Next, we observed that in the absence of MTZ‐induced injury, exogenous RA treatment did not alter Dendra2 fluorescence intensity or tooth mineralisation (Figure 4C–F). In contrast, when administered from R0D to R1D following MTZ ablation, exogenous RA significantly increased Dendra2 fluorescence intensity at both R0.5D and R1D in the injury group (Figure 4C–E). Moreover, alizarin red staining showed accelerated repair progression in both R3V and R5V compared to controls at R1D (Figure 4F). Among the 18 retinoid pathway genes upregulated by MTZ, qPCR analysis revealed that exogenous RA treatment significantly upregulated 8 genes (*crabp2a*, *crabp2b*, *cyp26b1*, *raraa*, *rarab*, *rxrab*, *rxrba* and *rxrbb*), while 10 genes (*rbp4*, *stra6*, *rbp1.1*, *lratb.1*, *lratb.2*, *rdh10a*, *dhrs3a*, *dhrs3b*, *aldh1a2* and *rarga*) showed no significant change (Figure 5A).

To further investigate the role of endogenous RA in tooth germ repair, zebrafish were treated with TZ, a selective CYP26A1/CYP26B1 inhibitor that prevents endogenous RA degradation [45]. Given that *raraa* showed the strongest upregulation in response to exogenous RA (Figure 5A), a dominant‐negative approach was used to generate *Tg*(*hsp70l:dnRARAA‐p2a‐DsRed; cryaa:venus*) transgene line [46, 47], which employs hsp70l heat‐shock promoter‐driven dominant‐negative RARAA mutation to disrupt RA downstream signalling. *cryaa:venus* was used to ensure that the existence of hsp70l recombinase was visible by the Venus fluorescence in the zebrafish eyes (Figure 5B,S,3A) [48, 49]. We found that TZ treatment suppressed *cyp26b1* expression at R1D (Figure S3B). Antibody staining and alizarin red staining showed that *Tg*(*hsp70l:dnRARAA‐p2a‐DsRed; cryaa:venus*) transgene line impaired the repair process after tooth germ injury, whereas TZ promoted it (Figure 5C–F).

However, exogenous addition of RA precursor substances (retinol or retinal) had no effect to the tooth germ repair process following injury (Figure S3C–F). Gene expression analysis by qPCR indicated that exogenous retinol enhanced *rbp4* expression, while retinal upregulated 6 genes (*rbp1.1*, *lratb.1*, *lratb.2*, *rdh10a*, *dhrs3a* and *dhrs3b*). Notably, neither retinol nor retinal affected *aldh1a2* expression (Figure S3G). These results suggest that RA, rather than retinol or retinal, is functionally distinct in regulating tooth germ repair after injury, with *aldh1a2* emerging as a key mediator within the RA signalling pathway during this process.

### 3.5 Modulation of *aldh1a2* Expression Regulate the Tooth Germ Injury Repair

**FIGURE 6:**
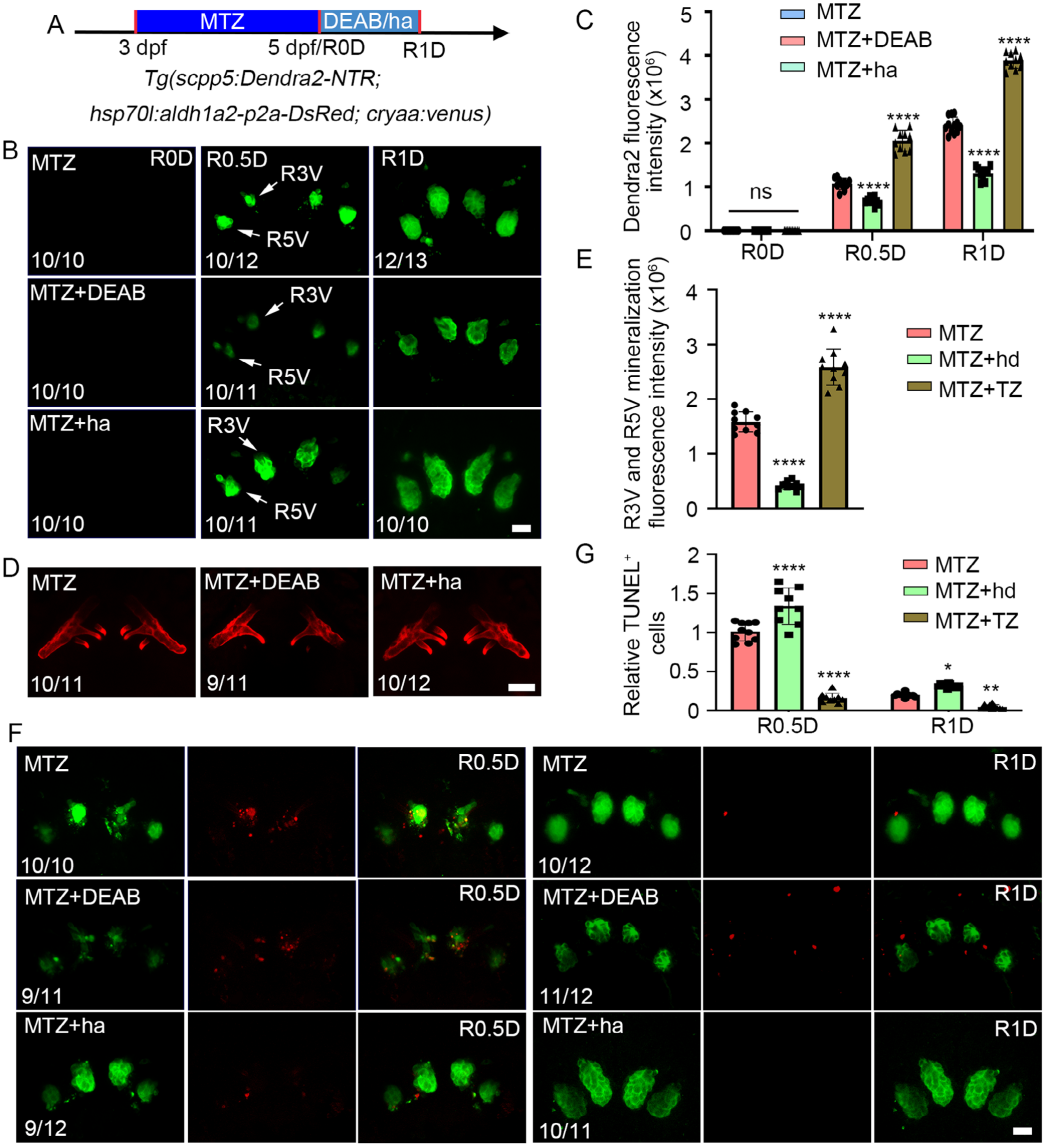
The role of aldh1a2 modulation in tooth repair. Experimental schedule of modulating *aldh1a2* (A). Antibody staining showing the Dendra2 fluorescence in MTZ, MTZ + DEAB and MTZ + ha groups from ROD to R1D. Scale bar is 20 μm (B, C). Alizarin red staining showing the tooth in MTZ, MTZ + DEAB and MTZ + ha groups at R1D. Scale bar is 50 μm (D, E). TUNEL staining showing the change of relative TUNEL^+^ cells at R0.5D and R1D in tooth germ cells. Scale bar is 20 μm (F, G). 4V^1^, The first generation‐tooth at position 4 in the ventral row; DEAB, 4‐diethylaminobenzaldehyde; dpf, days post‐fertilisation; ha, *Tg*(*hsp70l:aldh1a2‐p2a‐mCherry; cryaa:venus*); MTZ, Metronidazole; R0D, 0 day of repair; R3V, The repair‐tooth at position 3 in the ventral row. ns no significance, *p < 0.05, **p < 0.01 and ****p < 0.0001.

To investigate the functional role of *aldh1a2* in tooth germ repair after injury, we combined pharmacological inhibition with the specific aldehyde dehydrogenase inhibitor DEAB and genetic overexpression using the transgenic line *Tg*(*hsp70l:aldh1a2‐p2a‐mCherry; cryaa:venus*), which enables heat shock‐inducible expression of *aldh1a2* (Figures 6A and S3H) [49, 50]. DEAB treatment reduced, whereas overexpression of aldh1a2 increased, the expression of *aldh1a2* at R1D (Figure S3I). Antibody staining showed that DEAB significantly decreased Dendra2 fluorescence intensity at both R0.5D and R1D, while *aldh1a2* overexpression enhanced it (Figure 6B,C). Alizarin red staining indicated that DEAB impaired, while *aldh1a2* overexpression promoted, tooth germ repair at R1D (Figure 6D,E). TUNEL staining revealed opposing effects on apoptosis: DEAB increased the number of apoptotic cells, whereas *aldh1a2* overexpression decreased it in tooth germs at both time points (Figure 6F,G). Similarly, ISH analysis showed that DEAB downregulated, while *aldh1a2* overexpression upregulated, the expression of *cx43* and *fth1b* at R0.5D and R1D (Figure S4A,B). Consistent with these observations, qPCR analysis demonstrated that DEAB suppressed, and *aldh1a2* overexpression enhanced, the expression of genes involved in tooth germ cell differentiation and mineralisation (*ambn*, *col1a1a*, *col1a1b*, *enam*, *odam*, *runx2a*, *runx2b*, *scpp1*, *scpp8*, *scpp9*, *smad4a*, *sp7*, *spp1* and *vim*) (Figure S4C,D) [44, 51, 52]. Taken together, these results indicate that *aldh1a2* activity modulates the progression of tooth germ repair after injury.

## 4 Discussion

This study establishes that RA signalling plays an essential role in mediating tooth germ repair in zebrafish following NTR/MTZ‐induced injury. Specifically, after MTZ treatment, impaired mineralisation of 4V1 was observed at R0D, while 3V1 and 5V1 teeth were absent. This phenotypic pattern is explained by the earlier onset of SCPP5 expression in 4V1 compared to 3V1 and 5V1, and SCPP5 was first identified in the late cytodifferentiation phase of odontogenesis in 4V1 [39]. Consistent with this, SCPP5 fluorescence in the 4V1 region appeared before 3 dpf (Figure 1E). At 3.5 dpf, SCPP5 fluorescence was still detectable in the 4V1 of MTZ group, whereas no signal was yet present in the 3V1 and 5V1 regions of DMSO group (Figure S1C).

Although retinol, retinal and RA are sequential metabolites in the vitamin A pathway [53], they exert markedly different effects on zebrafish tooth germ repair. While exogenous RA promotes repair, retinol and retinal show no such activity. This functional divergence aligns with findings in other regeneration models: RA can induce regeneration in contexts such as the mouse ear pinna [54], rat alveolar [55] and frogs optic nerve axonal [56]. In the mouse ear pinna model, for instance, only RA—not retinol—supported complete regeneration of ear holes, likely because tissue injury suppresses the local RA synthesis pathway [54]. Similarly, in axolotl limb regeneration, retinol palmitate shows concentration‐dependent effects [57], and administration routes (local implantation vs. immersion) further modulate outcomes [58]. Moreover, retinol itself may exert biological effects independently of its metabolism into RA [59], whereas retinal displays dose‐dependent toxicity and pro‐apoptotic activity in adult retinal pigment epithelial cell line‐19 (ARPR‐19) [60]. In our zebrafish model, the inability of exogenous retinol and retinal to promote tooth repair may stem from two factors: insufficient local bioavailability at the tooth germ after intestinal absorption, and/or MTZ‐induced disruption of RA synthesis within dental cells.

RA signalling has been demonstrated to play crucial roles not only in governing tissue and organ development but also in facilitating repair and regeneration processes in specific tissues [54, 61]. In mammals, both excessive and deficient levels of RA can lead to severe abnormalities in tooth development [62, 63, 64]. However, modulation of RA signalling during zebrafish tooth development exhibited striking effects on tooth number patterning [18, 35, 36, 37, 38]. In post‐injury tooth repair or regeneration, the redifferentiation of stem cells is critical for preserving odontogenic potential. This process enables them to differentiate into essential cell types, including dental mesenchymal and epithelial cells [65, 66, 67]. Prior studies have established RA as an early ectodermal inducer that can direct the differentiation of pluripotent stem cells into dental epithelial lineages in vitro [68]. Furthermore, RA supplementation enhances the expression of odontogenic genes (e.g., Dmp1, Dlx2 and Spp1) and promotes the odontogenic capacity of cultured mouse dental mesenchymal cells [69]. Our findings demonstrate that RA treatment facilitated the repair of zebrafish R3V and R5V tooth germs. This effect may be attributed to RA‐enhanced redifferentiation of resident epithelial and mesenchymal stem cells or precocious development of tooth germ cells derived from 3V2 and 5V2. The complexity of RA signalling is reflected in its context‐dependent roles, as evidenced by variations across development, injury repair and different species.

In this study, targeted cell ablation was achieved using the NTR/MTZ system, in which NTR‐positive tooth germ cells convert MTZ into a cytotoxic compound, selectively inducing apoptosis [70]. During the subsequent repair phase in zebrafish, apoptosis in these cells is modulated by retinoic acid (RA) signalling—a pathway recognised for its critical role in balancing apoptosis and tissue remodelling during regeneration [71, 72]. Under basal conditions, transcriptional repressor complexes inhibit access to RA‐responsive genes. Upon nuclear entry and binding of RA to RAR/RXR heterodimers at RAREs, co‐activator complexes are recruited, leading to transcriptional activation of genes involved in cell fate determination, including those regulating differentiation and apoptosis [73]. A key characteristic of RA is its context‐dependent dual role in apoptosis, capable of either promoting or inhibiting cell death depending on variables such as cell type, retinoid species and coexisting signalling cues [72]. In various cancers, including oral [74], oropharyngeal [75], hepatic [76] and retinoblastoma [77], RA activation leads to growth arrest and apoptosis. Mechanistically, RA promotes apoptosis through multiple pathways, including regulation of caspase expression, modulation of pro‐ and anti‐apoptotic Bcl‐2 proteins, initiation of mitochondrial death pathways and activation of death ligand‐receptor signalling [72]. Conversely, in tissue repair and regeneration following injury, RA often exhibits an anti‐apoptotic function [72]. For example, in models of acute kidney injury, RA administration reduces cisplatin‐induced apoptosis, as indicated by decreased levels of cleaved caspase‐3 and fewer TUNEL‐positive renal epithelial cells [78]. Similarly, during fin regeneration in adult zebrafish, sustained RA signalling enhances the survival of proliferative blastemal cells via upregulation of the anti‐apoptotic protein Bcl‐2 [79]. Protective effects of RA have also been observed in epithelial and endothelial settings, such as mitigating TNFα‐induced apoptosis in lung epithelial cells and suppressing radiation‐induced apoptosis in keratinocytes and capillary endothelial cells [80, 81, 82]. Consistent with these findings, our study further supports a negative regulatory role for RA signalling in apoptosis during tooth germ repair following injury. Nevertheless, the precise molecular mechanisms underlying RA‐mediated regulation of apoptosis warrant further investigation.

## 5 Conclusion

Together, this work demonstrates the critical role of RA signalling in the repair of injured tooth germs. Our study supports this hypothesis through the establishment of a *Tg*(*scpp5:Dendra2‐NTR*) transgenic zebrafish line and the development of a targeted zebrafish tooth germ injury model using the MTZ/NTR system. Utilising this model, we observed that RA signalling regulates tooth germ repair. However, the interactions between RA signalling and other pathways, as well as its effects on epithelial–mesenchymal interactions, remain unclear and warrant further investigation. In conclusion, our findings offer novel insights into promoting tooth germ repair following injury.

## Author Contributions

**Qiqi Liu:** writing – review and editing, methodology, visualisation, investigation, data curation. **Zhenan Zhang:** writing – original draft, visualisation, validation, investigation. **Weifeng Hao:** visualisation, methodology. **Chunyan Zhou:** conceptualisation, validation. **Deqin Yang:** writing – review and editing, project administration, funding acquisition.

## Funding

This work was supported by the National Natural Science Foundation of China, 32270888, 31970783 and Start‐up Project of Introducing Talents to the Affiliated Stomatological Hospital of Fudan University/Shanghai Stomatological Hospital, SDC‐2024‐RC08.

## Ethics Statement

This present study was approved by the Ethics Committee of Stomatological Hospital of Chongqing Medical University (Approval No. 2022163).

## Conflicts of Interest

The authors declare no conflicts of interest.

## Supporting information


**Figure S1:** The role of MTZ in tooth injury: survival and apoptosis and RA signalling gene expression. The survival rate of zebrafish at R0D (A). Experimental schedule (B). TUNEL staining the relative TUNEL^+^ cells in DMSO and MTZ groups. Scale bar is 20 μm (C, D). 3V^1^, The first generation‐tooth at position 3 in the ventral row; dpf, days post‐fertilisation; MTZ, Metronidazole; R0D, 0 day of repair. ns no significance and ****p < 0.0001.

**Figure S2:** Effect of MTZ on the expression of *fth1b*, *cx43* and RA signalling‐related genes. ISH showing the expression of *fth1b* in DMSO and MTZ‐treated groups during the repair process. Scale bars: 100 μm (top) and 50 μm (bottom). (A). ISH showing the expression of *cx43* in DMSO and MTZ‐treated groups during the repair process. Scale bars: 100 μm (top) and 50 μm (bottom) (B). The of MTZ on the expression of RA signalling genes (C). MTZ, Metronidazole; R0D, 0 day of repair. ns no significance, *p < 0.05, **p < 0.01, ***p < 0.001 and ****p < 0.0001.

**Figure S3:** Two heat shock transgene lines and the effect of retinol and retinal on tooth germ repair. The *Tg*(*hsp70l:dnRARAA‐p2a‐DsRed; cryaa:venus*) transgene zebrafish line. Scale bar is 1000 μm (A). qPCR analysis of *cyp26b1* expression in MTZ and MTZ + TZ groups (B). Experimental schedule of exogenous retinol and retinal treatment (C). Antibody staining showing the Dendra2 fluorescence in MTZ, MTZ + Reo and MTZ + Rea groups. Scale bar is 20 μm (D, E). Alizarin red staining showing the tooth in MTZ, MTZ + Reo and MTZ + Rea groups at R1D. Scale bar is 50 μm (F). The effect of retinol and retinal on the expression of RA upstream genes (G). The *Tg*(*hsp70l:aldh1a2‐p2a‐mCherry; cryaa:venus*) transgene zebrafish line. Scale bar is 1000 μm (H). qPCR analysis of *aldh1a2* expression in the MTZ, MTZ + DEAB and MTZ + ha groups (I). 4V^1^, The first generation‐tooth at position 4 in the ventral row; DEAB, 4‐diethylaminobenzaldehyde; dpf, days post‐fertilisation; ha, *Tg*(*hsp70l:aldh1a2‐p2a‐mCherry; cryaa:venus*); MTZ, Metronidazole; R0D, 0 day of repair; R3V, The repair‐tooth at position 3 in the ventral row; Reo, retinol; Rea, retinal; TZ, talarozole. ns no significance, *p < 0.05, **p < 0.01, ***p < 0.001 and ****p < 0.0001.

**Figure S4:** Effects of modulating *aldh1a2* on tooth repair: examining *fth1b* and *cx43* expression, and key genes governing differentiation and mineralisation. ISH showing the expression of *fth1b* at R0.5D and R1D. Scale bars: 100 μm (top) and 50 μm (bottom) (A). ISH showing the expression of *cx43* at R0.5D and R1D. Scale bars: 100 μm (top) and 50 μm (bottom) (B). qPCR showing the expression of tooth germ cells differentiation and mineralisation gene at R0.5D (C). qPCR showing the expression of tooth germ cells differentiation and mineralisation gene at R1D (D). DEAB, 4‐diethylaminobenzaldehyde; ha, *Tg*(*hsp70l:aldh1a2‐p2a‐mCherry; cryaa:venus*); MTZ, Metronidazole; R0D, 0 day of repair. *p < 0.05, **p < 0.01, ***p < 0.001 and ***p < 0.001.

**Table S1:** The primer sequences of RNA probe.
**Table S2:** The primer sequences of qPCR.

## Data Availability

The data that support the findings of this study are available from the corresponding author upon reasonable request.
