# Supplementary figures and images for: Retinoic Acid Signalling Regulates Zebrafish Tooth Germ Repair Following Injury
Source: Cell Prolif. 2026 Feb 23;59(7):e70186. doi: 10.1111/cpr.70186 (PMC13325558; doi:10.1111/cpr.70186)

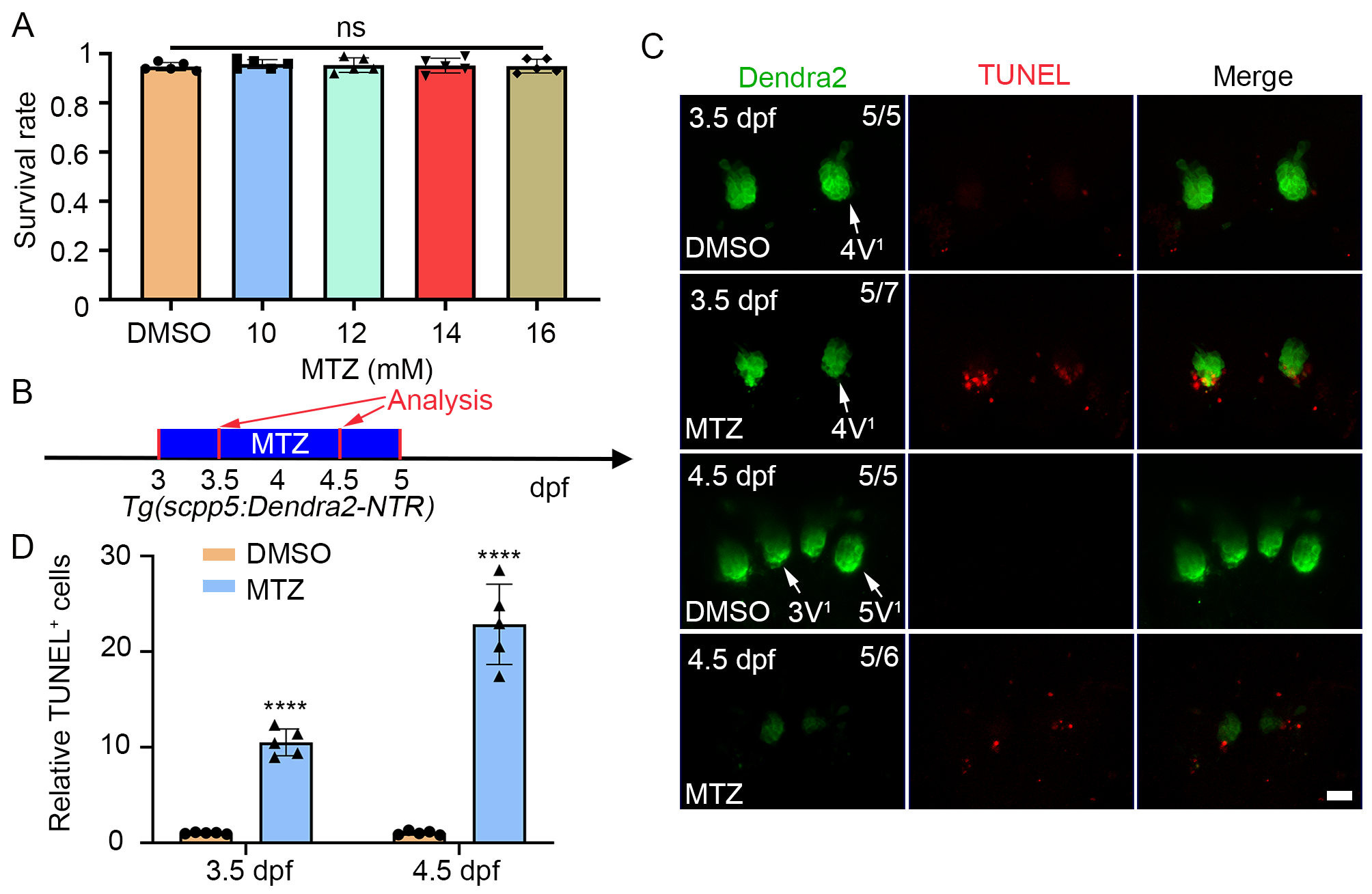

Supplement: Supplementary file 1 — Figure S1: The role of MTZ in tooth injury: survival and apoptosis and RA signalling gene expression. The survival rate of zebrafish at R0D (A). Experimental schedule (B). TUNEL staining the relative TUNEL+ cells in DMSO and MTZ groups. Scale bar is 20 μm (C, D). 3V1, The first generation‐tooth at position 3 in the ventral row; dpf, days post‐fertilisation; MTZ, Metronidazole; R0D, 0 day of repair. ns no significance and ****p < 0.0001. [file CPR-59-e70186-s002.tif]

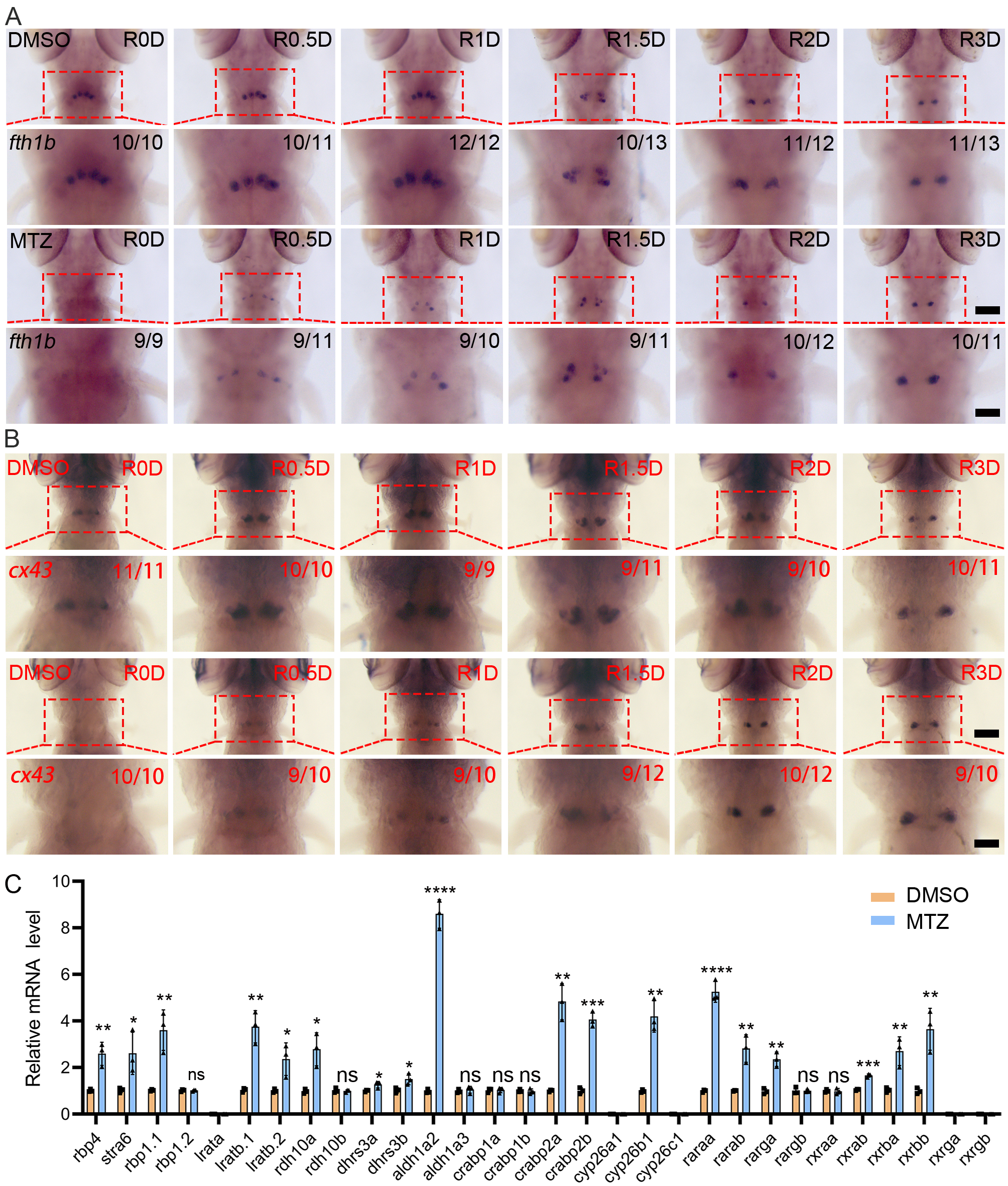

Supplement: Supplementary file 2 — Figure S2: Effect of MTZ on the expression of fth1b, cx43 and RA signalling‐related genes. ISH showing the expression of fth1b in DMSO and MTZ‐treated groups during the repair process. Scale bars: 100 μm (top) and 50 μm (bottom). (A). ISH showing the expression of cx43 in DMSO and MTZ‐treated groups during the repair process. Scale bars: 100 μm (top) and 50 μm (bottom) (B). The of MTZ on the expression of RA signalling genes (C). MTZ, Metronidazole; R0D, 0 day of repair. ns no significance, *p < 0.05, **p < 0.01, ***p < 0.001 and ****p < 0.0001. [file CPR-59-e70186-s005.tif]

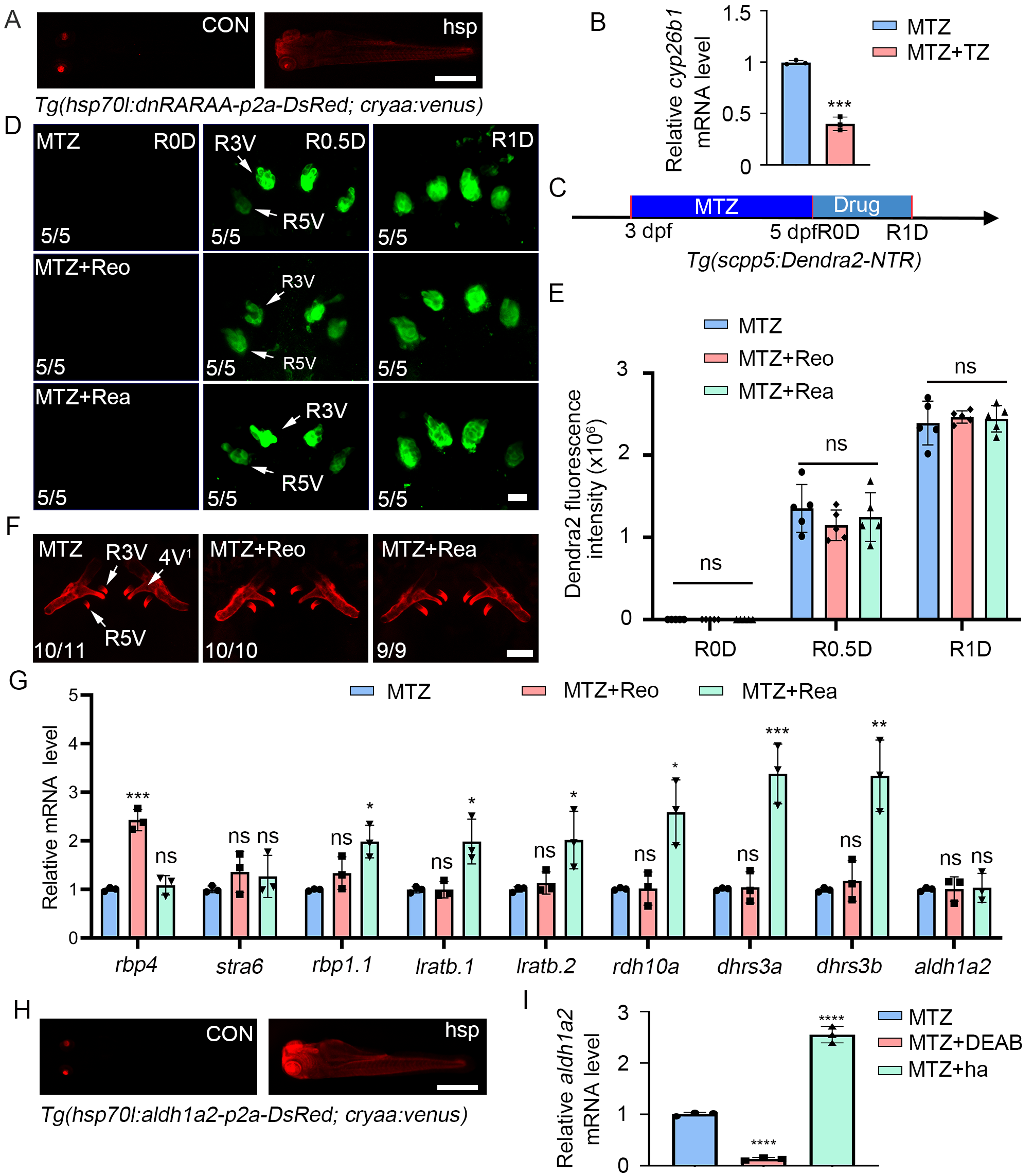

Supplement: Supplementary file 3 — Figure S3: Two heat shock transgene lines and the effect of retinol and retinal on tooth germ repair. The Tg(hsp70l:dnRARAA‐p2a‐DsRed; cryaa:venus) transgene zebrafish line. Scale bar is 1000 μm (A). qPCR analysis of cyp26b1 expression in MTZ and MTZ + TZ groups (B). Experimental schedule of exogenous retinol and retinal treatment (C). Antibody staining showing the Dendra2 fluorescence in MTZ, MTZ + Reo and MTZ + Rea groups. Scale bar is 20 μm (D, E). Alizarin red staining showing the tooth in MTZ, MTZ + Reo and MTZ + Rea groups at R1D. Scale bar is 50 μm (F). The effect of retinol and retinal on the expression of RA upstream genes (G). The Tg(hsp70l:aldh1a2‐p2a‐mCherry; cryaa:venus) transgene zebrafish line. Scale bar is 1000 μm (H). qPCR analysis of aldh1a2 expression in the MTZ, MTZ + DEAB and MTZ + ha groups (I). 4V1, The first generation‐tooth at position 4 in the ventral row; DEAB, 4‐diethylaminobenzaldehyde; dpf, days post‐fertilisation; ha, Tg(hsp70l:aldh1a2‐p2a‐mCherry; cryaa:venus); MTZ, Metronidazole; R0D, 0 day of repair; R3V, The repair‐tooth at position 3 in the ventral row; Reo, retinol; Rea, retinal; TZ, talarozole. ns no significance, *p < 0.05, **p < 0.01, ***p < 0.001 and ****p < 0.0001. [file CPR-59-e70186-s001.tif]

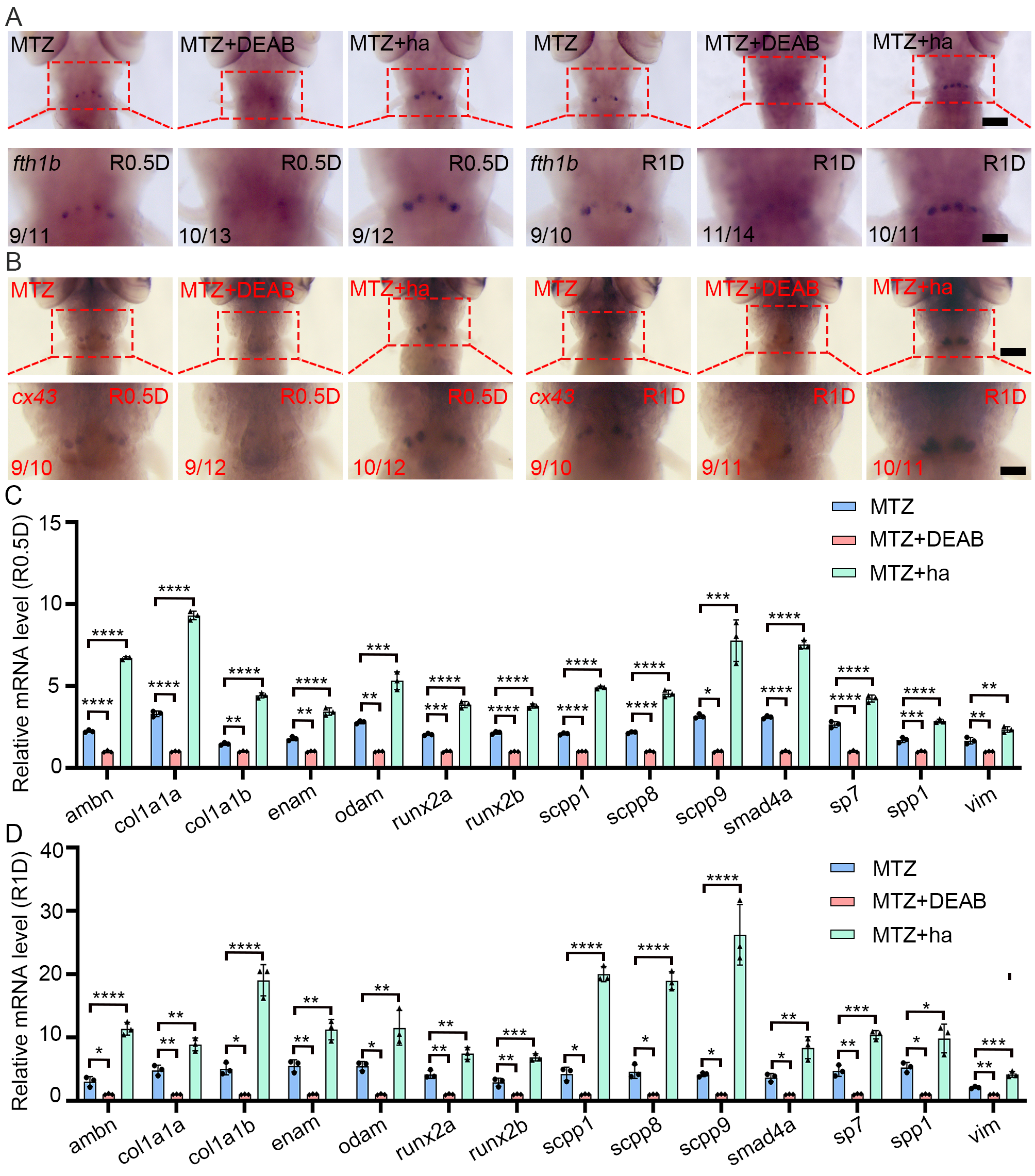

Supplement: Supplementary file 4 — Figure S4: Effects of modulating aldh1a2 on tooth repair: examining fth1b and cx43 expression, and key genes governing differentiation and mineralisation. ISH showing the expression of fth1b at R0.5D and R1D. Scale bars: 100 μm (top) and 50 μm (bottom) (A). ISH showing the expression of cx43 at R0.5D and R1D. Scale bars: 100 μm (top) and 50 μm (bottom) (B). qPCR showing the expression of tooth germ cells differentiation and mineralisation gene at R0.5D (C). qPCR showing the expression of tooth germ cells differentiation and mineralisation gene at R1D (D). DEAB, 4‐diethylaminobenzaldehyde; ha, Tg(hsp70l:aldh1a2‐p2a‐mCherry; cryaa:venus); MTZ, Metronidazole; R0D, 0 day of repair. *p < 0.05, **p < 0.01, ***p < 0.001 and ***p < 0.001. [file CPR-59-e70186-s003.tif]
