# Supplementary material for: Retinoic Acid Signalling Regulates Zebrafish Tooth Germ Repair Following Injury
Source: Cell Prolif. 2026 Feb 23;59(7):e70186. doi: 10.1111/cpr.70186 (PMC13325558; doi:10.1111/cpr.70186)
Supplement: Supplementary file 5 — Table S1: The primer sequences of RNA probe. Table S2: The primer sequences of qPCR. [file CPR-59-e70186-s004.docx]

**TABLE S1** The primer sequences of RNA probe.

| **Primers** | **Forward (5’–3’)** | **Reverse (5’–3’)** | **Accession number** | **Product size (bp)** |
| --- | --- | --- | --- | --- |

| *aldh1a2* | TTGGCTGATCTGGTGGAGAG | GAGGTCCGTGTTCAGTGGTT | NM_131850.1 | 760 |
| --- | --- | --- | --- | --- |
| *ambn* | CATCAACAGGTGCCCAATCC | GAGATGAAAGCTCCGTGACC | NM_001431095.1 | 731 |
| *cx43* | TGAGGTGGTCTTCCTGGTCA | GATGTGGCATGGCTAGACGT | NM_131038.1 | 664 |
| *enam* | AGAATGCTAGGACAGCTGCG | GGCTTGTCATCATCCCTCTC | NM_001145556.1_ | 638 |
| *fth1b* | ATGAGTTCTCAGGTGAGGCA | GAGTGTGTCTGTCGAACAGA | NM_001004562 | 514 |
| *scpp5* | ATGTGGACGTCTCTCCTGTG | GAGGAACTGTCTGTGGCATC | NM_001145236.1 | 406 |

**TABLE S2** The primer sequences of qPCR.

| **Primers** | **Forward (5’–3’)** | **Reverse (5’–3’)** | **Accession number** | **Product size (bp)** | |
| --- | --- | --- | --- | --- | --- |
| *aldh1a2* | ACAGTGCTTACCTTGCTACCC | CTTATCTGCCCATCCAGCGT | NM_131850.1 | | 122 |
| *aldh1a3* | AGGAGGCGGATAAAGCTGATG | TTGCCAGTGTCCTTCGACTC | NM_001044745.1 | | 181 |
| *ambn* | GCATTATAGCCGTGCCTCCT | GATGAAAGCTCCGTGACCTG | NM_001431095.1 | | 185 |
| *col1a1a* | CAGGAGCCCAGTGTTGAG | AGCCACCAGACATCTGAGGA | NM_199214.1 | | 173 |
| *col1a1b* | TGTATGGAAACCCGAGCCCTG | AGCAGGACCCACGGTATCAAC | NM_201478.1 | | 193 |
| *[crabp1a](https://zfin.org/ZDB-GENE-020320-3)* | AGCAGGACAATCCGAAGCTC | TGCTCTCCGTCCTGTCGAAT | NM_182858.1 | | 176 |
| *crabp1b* | GCTGGAACCTGGAAGATGAA | TTATCTCGGTGGTTCTGACG | NM_001001842.1 | | 181 |
| *[crabp2a](https://zfin.org/ZDB-GENE-020320-3)* | AGCTTTCCTCGCTGGGTAAC | CGTCAGCAGTCATGGTCAGAA | NM_182859.1 | | 133 |
| *[crabp2b](https://zfin.org/ZDB-GENE-020320-3)* | TACGGTGGATGGACGACCAT | TCGTTCATACACCCGAGTGC | NM_001320394.1 | | 187 |
| *cyp26a1* | AGTGGCCAGCATCAGTGAGAA | GAACGCCCTCATAATGGCCT | NM_131146.2_ | | 101 |
| *cyp26b1* | AGGGTATTCGAGCAAGAGAC | AGCTCAATAGTGGACTCCTTG | NM_212666.1 | | 175 |
| *cyp26c1* | ATGAACCACCAACTATTCTACCT | TTAGCGTTGCCACCTCACA | NM_001029951.2 | | 275 |
| *dhrs3a* | GTCGGGGATGTCACCATTCTT | GAGGCAGAAATGCTTTCGTGG | NM_001003477.1 | | 142 |
| *dhrs3b* | TGCATGGCAAGAGTTTGCTG | AAGGCTTTCGTGGTCCAAAAC | NM_001006070.1 | | 94 |
| *enam* | GGCTGTGGCAATGGAAATCG | CTCTAGCCAAGTCCCTGTGC | NM_001145556.1_ | | 214 |
| *lrata* | TTTTGTGGATGGCTGGTTGA | GTTGGACCGATGCATTTGAA | NM_001204131.2 | | 81 |
| *lratb.1* | TGCTGAACACGATGGACACT | ACTTTCGCAGAACTGGTCCG | NM_001440443.2 | | 97 |
| *lratb.2* | GAGCTGAGAAGCTCTGTGGG | CAGCTTTGCAGAACTGGTAGG | NM_001135971.1_ | | 123 |
| *odam* | CCTGAATACCGGGGTGATCG | GTCTCCTGTGAGGGATCTGC | NM_001145243.2 | | 126 |
| *raraa* | TTCACCACCCTTACCATCGC | CGTTGTGCATCTGTGTTCGG | NM_131406.2 | | 154 |
| *rarab* | TTGTGGGACTGACCCCTAGC | CTGGGTCTCCACAGAGTGAT | NM_131399.1 | | 158 |
| *rarga* | TGGACCCTTTTGCTTGGACC | AAGACTTGTCCTGGCAGACG | NM_131339.3 | | 147 |
| *rargb* | TCGTGTGCCAGGACAAATCT | GCGCGTCACTTTGTTGATCTG | NM_001083310.3 | | 146 |
| *rbp1.1* | AGACCTGGGTCCAGTTGATG | CAACCTCGCCTTTCTGGACA | NM_212895.2 | | 89 |
| *rbp1.2* | GCATGACAACTGTGAACTGG | ACCACTCCACAGACTCTCAT | NM_001114899.2 | | 130 |
| *rbp4* | TGGAACCATGACAGCCACC | CAGCAGCTCCCCAGTACTTC | NM_130920.2 | | 134 |
| *rdh10a* | AGTGCGTAAGGAGGTTGGTG | AAATGCCTTGGTGGTCCAGA | NM_001080583.2 | | 158 |
| *rdh10b* | GCCATGCTCACTTCTGGACTA | CAACCCCAAGGAGCTTGCTA | NM_201331.1 | | 95 |
| *runx2a* | CATGGTGGAGATCATAGCGGAC | CATTTCCAGCCATTACCGTCAC | NM_212858.2 | | 179 |
| *runx2b* | CAGACTTGAGTCCTTTCCCTGG | GGAGGTACGTGTGGTAATGAGC | NM_212862.2 | | 174 |
| *rxraa* | ATGGGCATGAAGAGAGAAGC | TGACTGGATCGTTGGGTGAA | NM_001161551.2 | | 205 |
| *rxrab* | TCTTCAAGAGGACGGTCAGA | CTCTTCATCCCCATGGCTAA | NM_131153.1 | | 133 |
| *rxrba* | TTCTCGGTCATCAGCTCATC | AAACCAAAGGGTGGCTTCAC | NM_001423448.1 | | 185 |
| *rxrbb* | CGGTGTCTCGATTACATGCT | TACACGCCATAATGCTTCCC | NM_131238.1 | | 151 |
| *rxrga* | TCTCAGTTCTTCGTTGCAGG | TGGACTGTTGAGTGCTCCAT | NM_131217.4 | | 208 |
| *rxrgb* | CCGGTCATGAGCTCTATGGT | GAGAGAGTTCATCTGTGGGC | NM_001002345.1 | | 190 |
| *scpp1* | TGACAGCCGACAACACTCAA | ACGACAACCTTTTCCTGGCT | NM_001145240.1 | | 196 |
| *scpp8* | TCCGAGTCAGATGAGAGCTC | GTTGAAGGCCTACGAGTAGG | NM_001145244.1 | | 104 |
| *scpp9* | CACTTCTGGAGTGAGAAACAGA | GCTGTCCTATAACCGCAGCA | NM_001145245.1 | | 216 |
| *smad4a* | CGGATCTGTCGGAGGAATCG | TCTTTAATGCTCTGCCGGGG | NM_001122700.2 | | 144 |
| *sp7* | GTACGGTAAAGCCTCGCATCTG | CGCTTATTGCACAGCAGACACG | NM_212863.2 | | 174 |
| *spp1* | ACAGACCACGCCAACAGAAT | TGATAATGGGACCCAGCGTG | NM_001002308.1 | | 188 |
| *stra6* | GTCAATCTGCTCATGCTCATGA | GACGGTCGGATACTCCTGTG | NM_001045312.1 | | 113 |
| *vim* | AAAGAGCAAAGTGGAGGTGGAC | TCTGCATCTCAGCAAGTTCCTC | NM_131872.2 | | 239 |
| *gapdh* | ACATGTTCCAGTACGACTCC | CTCTCGCTATAGACTGTGA | NM_001115114.1 | | 106 |
